# Toxicokinetic Study of a Gastroprotective Dose of Capsaicin by HPLC-FLD Method

**DOI:** 10.3390/molecules24152848

**Published:** 2019-08-05

**Authors:** Mónika Kuzma, Krisztina Fodor, Attila Almási, Gyula Mózsik, Tibor Past, Pál Perjési

**Affiliations:** 1 Institute of Pharmaceutical Chemistry, Faculty of Pharmacy, University of Pécs, Rókus str. 2, H-7624 Pécs, Hungary; 2 Department of Forensic Medicine, Medical School, University of Pécs, Szigeti str. 12, H-7624 Pécs, Hungary; 3 First Department of Medicine, Medical and Health Center, University of Pécs, Ifjúság str. 13, H-7624 Pécs, Hungary

**Keywords:** capsaicin, dihydrocapsaicin, non-steroid anti-inflammatory drugs, gastroprotection, high-performance liquid chromatography (HPLC), biotransformation

## Abstract

Background: A low dose of capsaicin and its natural homologs and analogs (capsaicinoids) have shown to prevent development of gastric mucosal damage of alcohol and non-steroid anti-inflammatory drugs. Based on this experimental observation, a drug development program has been initiated to develop *per os* applicable capsaicin containing drugs to eliminate gastrointestinal damage caused by non-steroid anti-inflammatory drugs. Methods: As a part of this program, a sensitive and selective reverse-phase high-performance liquid chromatography-based method with fluorescence detection has been developed for quantification of capsaicin and dihydrocapsaicin in experimental dog’s plasma. Results: The method was evaluated for a number of validation characteristics (selectivity, repeatability, and intermediate precision, LOD, LOQ, and calibration range). The limit of detection (LOD) was 2 ng/mL and the limit of quantification (LOQ) was 10 ng/mL for both capsaicin and dihydrocapsaicin. The method was used for analysis of capsaicin and dihydrocapsaicin in the plasma samples obtained after *per os* administration of low doses (0.1, 0.3, and 0.9 mg/kg bw) of Capsaicin Natural (USP 29) to the experimental animals. Conclusions: The obtained results indicated that the administered capsaicinoids did not reach the general circulation.

## 1. Introduction

**Figure 1:**
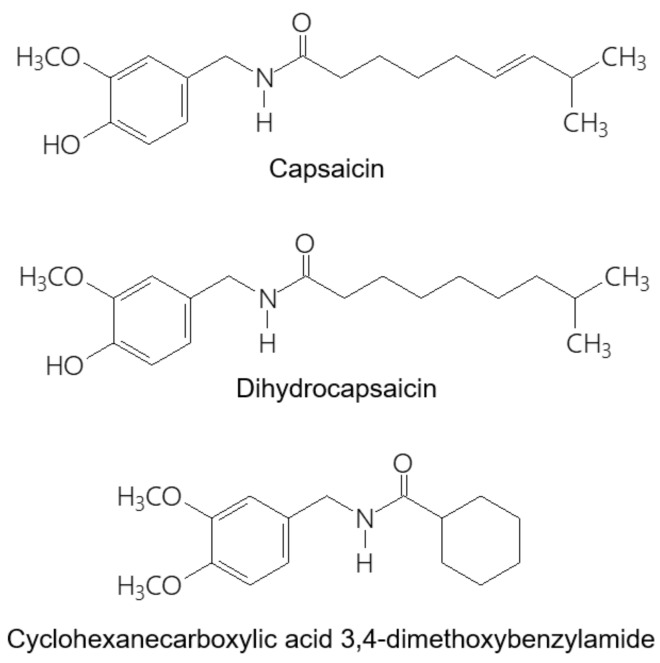
Chemical structures of capsaicin, dihydrocapsaicin and cyclohexanecarboxylic acid 3,4-dimethoxybenzylamide.

Capsaicinoids is the collective name of several structurally related compounds isolated from capsicum fruits. These substances produce the characteristic sensation associated with ingestion of spicy cuisine. Capsaicinoids include seven homologous branched-chain alkyl vanillylamides (capsaicin, dihydrocapsaicin, homocapsaicin I, homocapsaicin II, nordihydrocapsaicin, homodihydrocapsaicin I, homodihydrocapsaicin II) and three straight-chain analogs, octanoyl vanillylamide, nonoyl vanillylamide (nonivamide), and decoyl vanillylamide [1]. Among them, capsaicin and dihydrocapsaicin (Figure 1) are the most abundant compounds responsible for the pungency of the fruits. Capsaicinoids are used not only as a flavoring agent but also display several biological activities. Capsaicin functions as a high-affinity agonist of the TRPV1 receptor [2,3]. Most of the biological effects of capsaicin—and other capsaicinoids—are associated with activation of the capsaicin (TRPV1) receptor. However, some of the biological activities, like its anti-neoplastic and cardioprotective effects, have been found to be independent of the TRPV1 receptor [4].

It was earlier demonstrated that capsaicinoids display analgesic action [5,6,7,8,9], protect the gastric mucosa against the damage caused by the non-steroid anti-inflammatory drugs (NSAIDs) and alcohol [10,11,12,13,14,15], have anti-inflammatory effect [16,17,18], as well as anti-tumor and antioxidant potential [19,20,21]. Much of the published literature on capsaicin relates to capsaicin containing pepper extracts; these extracts are typically a mixture of capsaicin, dihydrocapsaicin, and the minor capsaicinoids nordihydrocapsaicin, homocapsaicin, and homodihydrocapsaicin [22]. The actual percentage of capsaicin and other capsaicinoids of the extracts varies depending on the peppers and the method of extraction. The structural characteristics of capsaicinoids responsible for their spicy flavor and biological activities (TRPV1 agonist activity) are the acid amide bond connecting a vanillyl ring and a fatty acid chain [3]. Several experiments showed the two main capsaicinoids (capsaicin and dihydrocapsaicin) to have comparable pharmacodynamic and pharmacokinetic properties [4,23,24,25,26,27].

Szolcsányi and Barthó were the first authors who clearly identified the beneficial and harmful effect of capsaicin in experimental peptic ulcer in the rats [10]. Later, a series of experiments proved that capsaicin introduced into the rat stomach in low concentrations effectively prevented gastric mucosal injury evoked by different harmful agents (e.g., aspirin, HCl, ethanol) [28,29,30,31,32]. Clinical studies with capsaicin started at the University of Pécs in 1997, which showed that (1) *per os* indomethacin produced a significant increase of gastric microbleeding in comparison to the controls; (2) *per os* capsaicin prevented in a dose-dependent manner of the indomethacin-induced gastric microbleeding in normal healthy human subjects; and (3) the gastroprotective effect of capsaicin on the indomethacin-induced gastric microbleeding remained the same after two weeks of capsaicin treatment. These observations proved that the capsaicin can prevent the ethanol- and the indomethacin-induced gastric mucosal damage in healthy human subjects [30,31,32].

Based on the clinical observations, a drug development program was launched at the University of Pécs to develop a *per os* applicable capsaicin containing drug to prevent gastrointestinal damage of non-steroid inflammatory drugs [14,15,32,33]. As a part of the program, pharmacokinetic and toxicological studies of capsaicinoids have been performed in the rat and the dog, respectively. Intestinal absorption and metabolism of capsaicinoids were investigated in rat proximal jejunum while 30 mg/mL standardized Capsicum extract (Capsaicin natural (Fluka)) was luminally perfused. It was found that both main capsaicinoids were fast absorbed from the jejunal loop. It was also demonstrated that the capsaicinoids were metabolized in the epithelial cells to the respective glucuronide conjugates, which were excreted back into the intestinal lumen [34].

The present work reports on the development and application of an HPLC method suitable for quantitation of capsaicin and dihydrocapsaicin in experimental dog’s plasma samples collected in the 28-day oral toxicity study of a standardized Capsicum extract Capsaicin Natural (United States Pharmacopeia (USP) 29) [14]. Although previous publications reported on intense hepatic metabolism of the *per os* administered capsaicinoids [4], as a part of the drug development program, a validated analytical method had to be developed to demonstrate the plasma concentration level of the administered capsaicinoids [14]. The method involves an HPLC-FLD analysis coupled with an automated solid-phase extraction, which gives good precision, accuracy, and recovery of the two capsaicinoids. The method is cost-effective, does not need MS instruments, but its sensitivity is comparable with that of the HPLC-MS methods previously reported [35,36,37,38]. The method was applied to the analysis of capsaicin and dihydrocapsaicin in dog’s plasma samples obtained after *per os* administration of standardized industrial Capsicum extract (Capsaicin Natural, USP 29) [39] to the experimental animals. The studies reported here were conducted according to the good laboratory practice (GLP) principles [40].

## 2. Methods and Materials

### 2.1. Chemicals and Reagents

USP Capsaicin reference standard (RS) and USP Dihydrocapsaicin reference standard (RS) were obtained from Bio-Separation Technologies (Budapest, Hungary). Cyclohexanecarboxylic acid 3,4-dimethoxybenzylamide (CADB) and phosphoric acid 85% were obtained from Sigma-Aldrich (Budapest, Hungary). Capsaicin natural standard (~65% capsaicin) was obtained from Fluka (Budapest, Hungary). Capsaicin Natural (USP 29) test item (used for the treatment of experimental animals) was purchased from Ashian Herbex Ltd. (Hyderabad, India). The capsaicinoid content of the extract was tested by the HPLC-DAD method according to the USP 29 requirements [39]. Important: Capsaicin and its natural and synthetic analogs can cause severe irritation, painful burning sensations, and uncontrollable cough.

HPLC-grade acetonitrile was obtained from Panreac Quimica Sa. (Barcelona, Spain). HPLC-grade isopropanol was produced by Carlo Erba Reagent Spa (Rodano, Italy). HPLC-grade methanol was obtained from J.T. Baker (Deventer, The Netherlands). Analytical reagent grade potassium hydroxide pellets were purchased from Merck Kraal (Darmstadt, Germany). Deionized water was purified in the Institute of Pharmaceutical Chemistry, the University of Pécs by use of a Millipore Direct-Q^TM^ system (Catalogue No.: PROG00002). Mobile phases used for HPLC were degassed in an ultrasonic bath (Realsonic cleaner) and filtered through ROBU glass filter (Por. 4) (ROBU Glasfilter Geraete, Hattert, Germany) before use.

### 2.2. HPLC Instrumentation and Chromatographic Conditions

The integrated high-performance liquid chromatography system (Agilent 1100; Agilent Technologies, Waldbronn, Germany)—which was qualified and verified according to the pharmaceutical requirements—was equipped with a quaternary pump, a degasser, an autosampler, an injector with a 100 μL loop, a column oven, an ultraviolet-visible, and a fluorescent detector. Data were recorded and evaluated using the Agilent ChemStation (Rev.A.10.02) software (Agilent Technologies, Waldbronn, Germany).

A binary gradient consisting of mobile phases A and B (A: 60% phosphate buffer (50 mM, pH 3.0, prepared by mixing orthophosphoric acid and potassium hydroxide solution) and 40% acetonitrile, B: 90% acetonitrile and 10% deionized water) was applied for the chromatographic separation. Separation of compounds was performed on a 4.6 mm × 150 mm, 5 µm particle size, ZORBAX Eclipse^®^ XDB-C8 analytical column (Agilent Technologies, Waldbronn, Germany) with guard cartridge (TR-C-160-K1; ABLE Jasco, Budapest, Hungary). Chromatography was performed at room temperature and the mobile phase flow rate was 1.5 mL/min. The compounds were separated with the following gradient profile: 0% B for 21 min, followed by a 5 min linear gradient to 95%, and finally a 10 min period at 95% B. The column was equilibrated to the initial conditions with a 5 min linear gradient to 0% B and an isocratic period of 15 min. Detection was fluorescent (λ_ex_ = 230 nm; λ_em_ = 323 nm). The injection volume was 25 μL for all sample solutions.

### 2.3. Preparation of the Standard Solutions

Stock solutions (1 mg/mL) of Capsaicin natural standard (Fluka), Capsaicin RS, Dihydrocapsaicin RS and stock solution (0.1 mg/mL) of cyclohexanecarboxylic acid 3,4-dimethoxybenzylamide (CADB) as internal standard (IS), were prepared in acetonitrile. The solutions were stored at −20 °C. Working standard solutions were prepared by dilution of the stock solutions with acetonitrile to give solutions of Capsaicin RS and Dihydrocapsaicin RS in the concentration range 10–500 ng/mL; these solutions were stored at 4 °C. System suitability solution was prepared by dissolving an accurately weighed quantity of Capsaicin RS, Dihydrocapsaicin RS and cyclohexanecarboxylic acid 3,4-dimethoxybenzylamide (CADB) in acetonitrile to obtain a solution having a known concentration of about 200 ng/mL of each component.

### 2.4. Toxicological Studies

The 28-day oral toxicity study of the USP Capsaicin natural was performed by the LAB International Research Centre Hungary Ltd. (Veszprém, Hungary). The study was also intended to provide toxicokinetic samples. Healthy beagle dogs (n = 8) of both sexes (32 dogs altogether) with an average weight of 7–12 kg were treated with *per os* administered Capsaicin Natural (USP 29) of four different dosages (0.0, 0.1, 0.3, and 0.9 mg/kg body weight/day) for 28 days. The standardized Capsaicin Natural (USP 29) [39] was administered in hard gelatin capsules Two types of capsules were used. The test item was weighed into a smaller capsule (Torpac, Fairfield, NJ, USA) and the smaller capsule containing the test item was placed into a bigger one (Capsula Operculata, Hungaropharma, Budapest, Hungary). The control animals were treated in the same manner orally with empty capsules [14]. Whole dog blood samples (4.5 mL) were collected in pre-labeled S-Monovette Lithium-Heparin Gel+ vacutainer tubes (Sarstedt, Nümbrecht, Germany), before the treatment (0.0 h) and after the treatment at the 0.25, 0.5, 1.0, 2.0, 3.0, and 4.0-h timepoints, on the first and the last day of the treatment. The blood samples were centrifuged, plasma and erythrocytes were separated and stored at −70 °C until analysis.

The study (Study code: 07/000-100K; LAB International Research Centre Hungary Ltd., Veszprém, Hungary) was performed in compliance with the principles of good laboratory practice regulations [40] and the FDA 21 CFR, Part 58 (Good Laboratory Practice for Nonclinical Laboratory Studies) [41]. (Basis of study: Guideline on repeated dose toxicity, CPMP/SWP/1042/99 (London, 16 December 1999. [42])

### 2.5. Sample Preparation

#### 2.5.1. Plasma

Separated dog plasma samples stored at −70 °C were defrosted keeping them in 35 °C water bath. In a 1.5 mL centrifuge tube, a 500 µL aliquot of the plasma was spiked with 10 μL of cyclohexanecarboxylic acid 3,4-dimethoxybenzylamide (CADB) solution (1.0 μg/mL). After vortex mixing, 500 μL cold acetonitrile was added to the tubes and vortex mixed for 20 s. After centrifugation for 10 min at 15,000× g, the supernatant was transferred to another centrifuge tube, mixed with 250 μL of deionized water and vortex mixed for 20 s. Then the sample was transferred onto an SPE cartridge (AccuBOND II ODS-C18 cartridges, 200 mg, 3 mL; Agilent Technologies), which was previously conditioned with methanol (2 mL) and water (2 mL). After addition of the sample, the cartridge was washed with water (1 mL) and with methanol (1 mL). Flow speed was 1 mL/min (in both cases). The collected methanol solution was evaporated to dryness by using nitrogen gas. The sample was stored in a deep freezer at −70 °C until analysis. Before analysis, the dry plasma extract was reconstructed in 50 μL of acetonitrile.

#### 2.5.2. Erythrocytes

Separated dog erythrocyte samples stored at −70 °C were defrosted keeping them in 35 °C water bath. Erythrocytes were suspended in 0.1 M phosphate buffer (pH 7.2) by vortex mixing to the volume of the original whole blood samples. The obtained samples were sonicated for 15 min at room temperature, vortexed for 20 s, and centrifuged at 450× *g* for 15 min. Then, 500 µL of the supernatants were extracted in the same way as the plasma samples described in 2.5.1. Before analysis, the obtained extracts were reconstructed in 50 μL of acetonitrile.

## 3. Results

### 3.1. Extraction of the Plasma Samples

Sample preparation is a critical step for accurate and reliable bioanalytical assay. The most widely employed bioanalytical sample preparation methodologies currently are the liquid–liquid extraction (LLE), protein precipitation (PPT), and the solid-phase extraction (SPE). Due to the strong protein-binding character of capsaicin [2], initially, the PPT method was applied using acetonitrile. Since neither the purity of the extracts nor the recovery of the compounds could reach the satisfactory level at the selected concentration range, the combination of the PPT and the SPE methods has been applied.

Accordingly, the applied chromatographic internal standard should serve as an extraction standard at the same time. CABD, based on its similar structure to the capsaicinoids, seemed to an appropriate substance to fulfill both aims. Although the retention time of the selected compound turned not to be optimal for the HPLC analysis, statistical analyses gave acceptable validation parameters while using it. The above extraction experiments proved that the recovery properties of CADB are similar to the two capsaicinoids, so we could use CADB as both an internal standard and extraction standard in optimal concentration, e.g., the system suitability solution contains the same concentration for Capsaicin RS, Dihydrocapsaicin RS, and CABD. (Table 1 and Table 2)

### 3.2. Extraction of the Erythrocytes

To test if capsaicinoids are present in the blood cells or bound to them, the separated erythrocytes were reconstructed in 0.1 M phosphate buffer (pH 7.2) and disintegrated by sonication, according to the method successfully applied for several acidic, neutral, and basic drugs [44]. The obtained supernatants were extracted and analyzed similarly to the plasma samples. Control recovery experiments using Capsaicin RS, Dihydrocapsaicin RS, and CABD were performed using human blood (plasma and erythrocytes, separately) as described in 2.4., 2.5.1, and 2.5.2. The recovery data were found to be similar to those summarized in Table 3.

### 3.3. Method Validation

#### 3.3.1. Specificity

**Figure 2:**
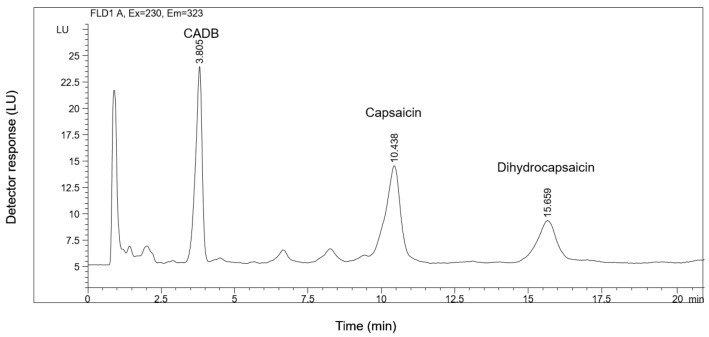
HPLC-FLD chromatogram of the extract of a blank dog plasma spiked with Capsaicin natural standard (Fluka; c = 50 ng/mL) and cyclohexanecarboxylic acid-3,4-dimethoxybenzylamide (CADB) as internal standard (IS) (c = 20 ng/mL).

**Figure 3:**
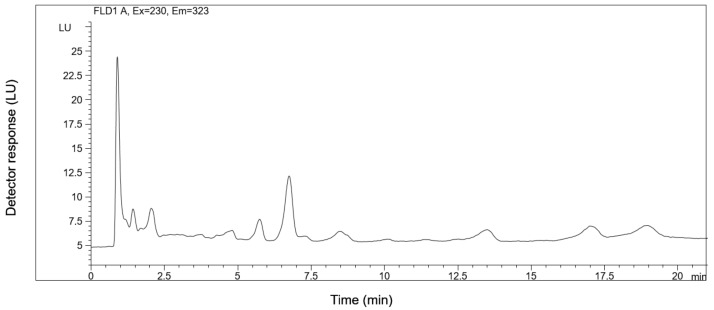
HPLC-FLD chromatogram of the extract of a blank dog plasma sample.

Specificity tested the ability of the method to differentiate and quantify the analytes in the presence of endogenous constituents in the sample. The HPLC-FLD chromatogram of a dog plasma spiked with Capsaicin natural standard (Fluka, c = 50 ng/mL) and cyclohexanecarboxylic acid 3,4-dimethoxybenzylamide (CADB) (c = 20 ng/mL) is shown in Figure 2. The retention times of capsaicin, dihydrocapsaicin, and CADB were 10.44, 15.66, and 3.81 min, respectively. Figure 3 shows an HPLC chromatogram of a blank dog plasma sample indicating no endogenous peaks at the retention times of capsaicin, dihydrocapsaicin, and CADB.

#### 3.3.2. Accuracy

Accuracy was calculated by spiking control plasma samples with the accurate amount of Capsaicin RS, Dihydrocapsaicin RS of 10 ng/mL, 20 ng/mL, and 40 ng/mL, and CADB of 20 ng/mL plasma concentration. After the solid-phase extraction of the samples, the percentage of recoveries was calculated. The evaluation was based on the relative standard deviation (RSD%) (Table 1).

#### 3.3.3. Linearity

Linearity was studied by preparing standard solutions and control dog’s plasma samples spiked with Capsaicin RS, Dihydrocapsaicin RS, and CADB at different concentrations from 10 to 500 ng/mL and determining the linearity by least-squares regression. Using the standard solutions, the method was linear in the range of 10–500 ng/mL for capsaicin (y = 1.0395x−0.3753, where y is peak area and x is concentration (ng/mL), r^2^ = 0.9984), for dihydrocapsaicin (y = 1.0458x− 1.4624; r^2^ = 0.9997), and for CADB (y = 1.4315x−0.7703; r^2^ = 0.9996) as well. Data were obtained at 5 levels of concentration (10; 20, 50; 100; 500 ng/mL) from 5 parallel injections of 2 independent weightings of the substances.

Control plasma samples were spiked with CADB (c_plasma_ = 20 ng/mL) and different amounts of Capsaicin RS and Dihydrocapsaicin RS, and ratios of peak areas are investigated. Data were obtained at 5 levels of plasma concentration (10; 20, 50; 100; 500 ng/mL) from 3 independent weightings of the substances. The method was linear in the range of 10–100 ng/mL for both Capsaicin RS (y = 0.0441x−0.00002, where y is peak area and x is concentration (ng/mL); r^2^ = 0.9979) and for Dihydrocapsaicin RS (y = 0.0458x + 0.0107; r^2^ = 0.9992).

#### 3.3.4. System Suitability

System suitability data and system precision were evaluated based on the chromatograms of (1) solutions containing Capsaicin RS, Dihydrocapsaicin RS, and cyclohexanecarboxylic acid 3,4-dimethoxybenzylamide (CADB) (c = 200 ng/mL, in acetonitrile, each), and (2) solutions containing Capsaicin natural (Fluka; c = 500 ng/mL, in acetonitrile) and (3) CADB (c = 200 ng/mL, in acetonitrile). Results were obtained from 6 parallel injections. The evaluation was based on the relative standard deviation (RSD%). System suitability data and system precision data are summarized in Table 2 and Table 3.

#### 3.3.5. Precision

Precision was studied by investigating repeatability and intermediate precision. Repeatability was determined by measuring intra-day data of 3 parallel injections of 3 parallel dilutions of 2 independent weighings of Capsaicin RS, Dihydrocapsaicin RS, and cyclohexanecarboxylic acid 3,4-dimethoxybenzylamide (CADB) (c = 200 ng/mL, in acetonitrile, each). Intermediate precision was determined by measuring inter-day (by injection of the samples over three consecutive days) data of 3 parallel injections of 3 dilutions (from 2 weighings) of Capsaicin RS, Dihydrocapsaicin RS, and Cyclohexanecarboxylic acid 3,4-dimethoxybenzylamide (CADB) (c = 200 ng/mL, in acetonitrile, each). The evaluation was based on the relative standard deviation (RSD%) (Table 4 and Table 5).

#### 3.3.6. Matrix Effect

Matrix effect was studied after solid-phase extraction of blank dog plasma. After evaporating the extract to dryness, the residue was dissolved in a solution containing Capsaicin RS (20, 100, 200, and 400 ng/mL), Dihydrocapsaicin RS (20, 100, 200, and 400 ng/mL) and cyclohexanecarboxylic acid 3,4-dimethoxybenzylamide (CADB) (c = 200 ng/mL) in acetonitrile (Table 6). The evaluation was performed by ANOVA for solutions containing Capsaicin RS, Dihydrocapsaicin RS, and cyclohexanecarboxylic acid 3,4-dimethoxybenzylamide (CADB) dissolved in acetonitrile containing the dried solid-phase extracts and pure acetonitrile. F_calculated_ was smaller than F_critical_ for each compound at each investigated concentration (Table 7).

#### 3.3.7. Determination of LOD and LOQ

The limit of detection (LOD) (3 times baseline noise) and the limit of quantification (LOQ) (10 times baseline noise) were determined visually based on the signal-to-noise approach. The LOD and LOQ values of capsaicin and dihydrocapsaicin in dog’s plasma were found to be 2 ng/mL and 10 ng/mL, respectively.

## 4. Discussion

The present work was planned and accomplished as a part of the drug development program initiated by the Department of Pharmacology and the First Department of Medicine of the University of Pécs. As part of the drug development program, a complex toxicological investigation was performed by the LAB International Research Centre Hungary Ltd. As part of the toxicological investigations, toxicokinetic studies were performed in cooperation of the LAB International Research Centre and the GLP Laboratory of the Institute of Pharmaceutical Chemistry of the University of Pécs [14].

The present contribution describes development of an HPLC-FLD method with combined protein precipitation (PPT) plus solid-phase (SPE) extraction, and its application to quantify the main capsaicinoids, capsaicin, and dihydrocapsaicin, in dog’s plasma samples. The method was validated by specificity, accuracy, linearity, system suitability, precision, matrix effect, LOQ, and LOD. The limit of quantification (the lowest capsaicin and dihydrocapsaicin concentration at which percent error and RSD were < 15%) was 10 ng/mL for both compounds. The limit of detection for both capsaicin and dihydrocapsaicin extracted from plasma by solid-phase extraction method was 2.0 ng/mL plasma concentration. The recovery percentages of the solid-phase extraction were found 78.52% for capsaicin and 86.30% for dihydrocapsaicin. These values are higher than those of the extraction method used by Donnerer et al. while analyzing rat blood samples [45]. The detector response was linear over the range of 10–500 ng/mL plasma concentration of both capsaicin and dihydrocapsaicin. The sensitivity of the present method is comparable with that of the HPLC-MS methods previously reported for determination of the two main capsaicinoids in rat plasma and tissues [35,36,37,38].

In the in vivo experiments, healthy beagle dogs were treated with *per os* administered Capsaicin Natural (USP 29) of four different doses (0.0, 0.1, 0.3, or 0.9 mg/kg body weight/day) for 28 days. The applied test item was qualified by the HPLC-DAD method. Its capsaicin and dihydrocapsaicin contents were 61.32% and 22.59%, respectively [39]. During the toxicological study, no adverse effect of the applied doses of the Capsaicin Natural test item was observed in either sex group [14].

**Figure 4:**
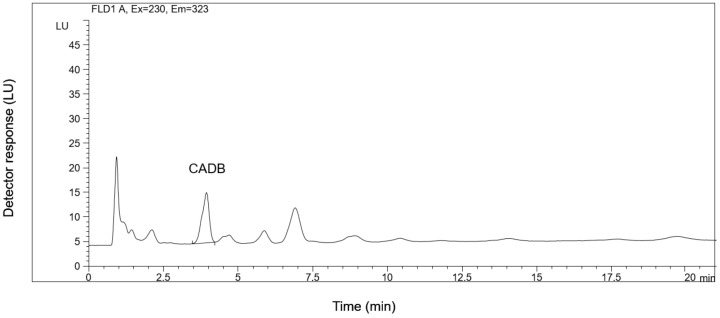
HPLC-FLD chromatogram of the extract of the plasma of an experimental dog 60 min after *per os* administration of Capsaicin Natural (USP 29, 0.9 mg/kg) test item, spiked with cyclohexanecarboxylic acid-3,4-dimethoxybenzylamide (CADB) as internal standard (IS) (c = 20 ng/mL).

Blood samples were collected at the 0.25, 0.5, 1.0, 2.0, 3.0, and 4.0-h timepoints on the first and the last day of the treatments. Analysis of the samples showed that neither capsaicin nor dihydrocapsaicin could be detected even in the case of administration of the highest applied dose (Figure 4). These observations were similar in both sex groups of the experimental animals. It is worth mentioning that based on the average plasma volume of the used 7–9-month-old (9 kg average body weight) experimental beagle dogs [46], the peak plasma concentration (iv. administration; 100% bioavailability; 100% recovery from the blood) of the capsaicin content of the Capsaicin Natural USP 29 test item would reach as high as 12.46 μg/mL. This concentration is four magnitudes higher than the LOD (10 ng/mL) of the used method. The HPLC-FLD analysis results were confirmed by investigation of the plasma samples by HPLC-MS as well [13,14]. Chromatograms of the analyzed plasma samples of the experimental animals treated with 0.9 mg/kg Capsaicin Natural at the 0.0, 0.25, 0.5, 1.0, 2.0, 3.0, 4.0-h timepoints are provided as Supplementary Materials (Figures S1–S7). The chromatograms are those obtained by analysis of the samples of the first day after the treatments.

These results draw attention to some previous studies related to the pharmacokinetics of capsaicinoids. For example, intragastrically administered capsaicin to the rat had minimal immediate blood pressure responses [47] in comparison to that one observed in the case of intravascular or subcutaneous [48]. Other investigations have provided evidence that low doses of capsaicinoids are effectively absorbed from the small intestine of the rat [4,24,34,49,50]. Furthermore, capsaicinoids, when administered to rats intragastrically, were readily absorbed from the gastrointestinal tract and were metabolized to a great extent in the liver before reaching the general circulation [4,45]. Capsaicinoids are metabolized to a great extent by the hepatic enzymes in the rat [26] and the dog [45,49,50]. The most abundant hepatic metabolites were 16-hydroxycapsaicin, 17-hydroxycapsaicin, and 16,17-dehydrocapsaicin [4,14,26,45,49,50]. In addition to these three metabolites, rat microsomes also produced vanillylamine and vanillin [4,26,51,52]. The specificity of capsaicinoids’ binding to TRPV1 receptor is based on the interaction of the vanillyl moiety and the acid amide bond. The aliphatic chain develops nonspecific interactions with the channel of the receptor [9]. Accordingly, the non-hydrolized, chain-modified metabolites (like capsaicin and dihydrocapsaicin) are expected to display similar TRPV1-based biological effects [2,3,9].

The standardized capsicum extract (Capsaicin Natural (USP 29) was administered in double-layered hard gelatin capsules. The disintegration time of such capsules in the dog stomach was found to be about 20 min [53]. Accordingly, release of the test item from the double-layered capsule needed about 30–40 min. Previously, *per os* administration of fresh capsicum (with 26.6 mg capsaicin content) to healthy adult volunteers resulted in 2.47 + 0.13 ng/mL peak plasma concentration (C_max_) and T_max_ 47.08 + 1.99 min. This treatment corresponds to 0.38 mg/kg bw capsaicin dose (70 kg average body weight). The absorbed capsaicin was rapidly metabolized (T_1/2_ 24.87 + 4.97 min). It was detectable in the plasma starting at 15 min until 90 min. The authors explained the low C_max_ by an intensive metabolism of the absorbed capsaicin in the liver [54].

In another experiment, pharmacokinetics of *per os* administered capsaicin (30 mg/kg bw; suspended in refined peanut oil) was studied in the rat. The maximum amount of the detectable capsaicin (24.4%) in the investigated organs and tissues was found at the 1-h timepoint, which was reduced to 1.24% in 24 h. By far the highest amounts of the absorbed capsaicin were found in the intestine and the liver over the first six hours. The plasma peak concentration (1,9 μg/mL) was found at the 1-h timepoint [49]. These latter experiments gave evidence of accumulation of the absorbed capsaicinoids in the intestinal and hepatic cells. Combined with the previously reported intense hepatic metabolism, this latter finding can rationalize the low plasma peak concentration of the parent compounds. (The intestinally accumulated capsaicinoids are released over an extended period of time, reducing the peak concentration and making the hepatic metabolism more effective.) These experimental data are in accordance with our results indicating that in the case of administration of a low dose of capsaicinoids (Capsicum extracts), even the major capsaicinoids do not reach the central circulation at a detectable level.

After recognizing the lack of capsaicinoids in the analyzed plasma samples, an experiment using an extra high single dose (4.8 mg/kg) of Capsaicin Natural (USP 29) was performed using two experimental animals. Analysis of the plasma samples of this latter experiment did not show presence of either capsaicin or dihydrocapsaicin. Chromatogram of the analysis of the plasma samples collected at the 0.0-h (Figure S8) and the 2.0-h (Figure S9) timepoints are provided as Supplementary Materials. (In this experiment 2.0 h was the last timepoint of blood sampling.)

Since several drugs have been reported to accumulate in the blood cells or bound to them [44], we have performed an analysis of the separated erythrocytes as well. In these experiments, the separated dog blood cells were resuspended in phosphate buffer (pH 7.2), disintegrated by sonication [44] and the supernatants were analyzed by the validated HPLC-FLD method. Similar to the results obtained for the corresponding plasma samples, neither capsaicin nor dihydrocapsaicin could be detected in the extracts. Control recovery experiments using Capsaicin RS, Dihydrocapsaicin RS, and CABD were performed using human blood (plasma and erythrocytes, separately) as described in 2.4., 2.5.1, and 2.5.2. The recovery data were found similar to those summarized in Table 1.

Based on the above results, development of a family of combinational (low dose capsaicin plus NSAID) products has been started that has industrial property protection. [55]. By this time, the Phase I study has been completed in the First Department of Medicine, Medical and Health Center, University of Pécs (Pécs, Hungary). The preliminary results are in accordance with the above preclinical results, neither capsaicin nor dihydrocapsaicin could be detected in the extracts.

## 5. Conclusions

A sensitive and selective reverse-phase high-performance liquid chromatography (RP HPLC) method with fluorescent detection (FLD) has been developed for quantification of the two main capsaicinoids (capsaicin and dihydrocapsaicin) in dog’s plasma. The plasma samples were obtained from experimental dogs treated with different doses of *per os* applied Capsaicin natural (USP 29). Analysis of the two capsaicinoids in the experimental animals’ plasma indicated that the parent compounds could not be detected in the samples even in the case of the highest *per os* dose. The results are in accordance with the earlier results demonstrating that intestinal accumulation and hepatic metabolism limit the systemic pharmacological effects of the enterally absorbed capsaicin.

## Figures and Tables

**Table 1:** Data for system suitability and system precision of acetonitrile solutions of Capsaicin RS (200 ng/mL), Dihydrocapsaicin RS (200 ng/mL), and cyclohexanecarboxylic acid-3,4-dimethoxybenzylamide (CADB) as internal standard (IS) (200 ng/mL) [43].

Injections (Standard Solutions)	CADB		Capsaicin RS		Dihydrocapsaicin RS	
	t_R_(min)	Area	t_R_(min)	Area	t_R_(min)	Area
1	3.766	284.117	10.374	202.757	15.571	190.972
2	3.769	284.072	10.382	200.816	15.586	191.821
3	3.769	284.350	10.391	201.201	15.598	189.120
4	3.769	285.569	10.392	200.070	15.596	190.495
5	3.774	283.781	10.397	201.964	15.597	190.939
6	3.769	283.805	10.390	200.689	15.599	189.844
Mean	3.769	284.282	10.388	201.250	15.591	190.532
RSD%	0.068	0.234	0.080	0.481	0.070	0.497
**Compounds**	**t_R_(min)**	**RRT**	**k′**	**T**	**N**	**R_s_**
CADB	3.769	-	2.91	1.29	1109	-
Capsaicin	10.388	2.76	9.79	1.26	1946	9.48
Dihydrocapsaicin	15.591	4.14	15.19	1.20	2822	4.91

t_R_: retention time; RRT: relative retention time (RRT = t_R(*i*)_/t_R(ref)_; where t_R(*i*)_ and t_R(ref)_ are the retention times of component *i* and the reference compound); k’: capacity factor; T: asymmetry factor; N: number of theoretical plates; R_s_: resolution.

**Table 2:** System suitability and system precision data of a solution containing Capsaicin natural (Fluka; 500 ng/mL) and internal standard (CADB) (200 ng/mL) in acetonitrile [43].

Injections (Standard Solutions)	CADB		Capsaicin RS		Dihydrocapsaicin RS	
	t_R_(min)	Area	t_R_(min)	Area	t_R_(min)	Area
1	3.788	286.467	10.364	285.920	15.534	168.890
2	3.790	286.425	10.371	285.133	15.551	170.850
3	3.799	286.970	10.395	285.046	15.595	172.887
4	3.796	287.513	10.390	287.439	15.570	172.632
5	3.799	287.942	10.400	287.673	15.608	172.359
6	3.798	286.939	10.403	288.441	15.599	172.231
Mean	3.795	287.043	10.387	286.609	15.576	171.642
RSD%	0.127	0.207	0.154	0.500	0.189	0.887
**Compounds**	**t_R_(min)**	**RRT**	**k′**	**T**	**N**	**R_s_**
CADB	3.795	-	3.10	1.49	1453	-
Capsaicin	10.387	2.74	10.23	1.44	2486	10.69
Dihydrocapsaicin	15.576	4.10	15.84	1.25	3121	5.32

t_R_: retention time; RRT: relative retention time (RRT = t_R(*i*)_/t_R(ref)_; where t_R(*i*)_ and t_R(ref)_ are the retention times of component *i* and the reference compound); k’: capacity factor; T: asymmetry factor; N: number of theoretical plates; R_s_: resolution.

**Table 3:** Data for accuracy of determination of Capsaicin RS, Dihydrocapsaicin RS, and cyclohexanecarboxylic acid-3,4-dimethoxybenzylamide (CADB) as internal standard (IS) in blank dog plasma.

CADB			Capsaicin RS			Dihydrocapsaicin RS		
c_spiked plasma_ (ng/mL)	Area	Recovery %	c_spiked plasma_ (ng/mL)	Area	Recovery %	c_spiked plasma_ (ng/mL)	Area	Recovery %
20	191.35	67.11	10	77.42	74.84	10	92.86	91.73
20	218.95	76.75	10	86.85	83.91	10	97.98	96.64
20	186.92	65.56	10	84.02	81.19	10	76.49	76.03
20	222.89	78.12	10	81.84	79.09	10	89.01	88.04
20	211.78	74.24	20	171.74	82.79	20	183.61	89.39
20	190.08	66.66	20	159.88	77.08	20	180.82	88.05
20	166.62	58.47	20	134.45	64.85	20	152.89	74.66
20	191.31	67.09	20	130.54	62.97	20	157.25	76.75
20	184.59	64.74	40	348.6	83.93	40	368.06	88.93
20	216.36	75.84	40	354.28	85.29	40	367.99	88.91
20	192.02	67.34	40	353.13	85.02	40	352.48	85.19
20	177.84	62.39	40	344.44	82.93	40	349.48	84.47
**Mean recovery %**		**68.69**			**78.52**			**86.30**
**RSD %**		**8.956**			**9.888**			**8.101**

**Table 4:** Repeatability data for acetonitrile solutions of Capsaicin RS, Dihydrocapsaicin RS, and cyclohexanecarboxylic acid-3,4-dimethoxybenzylamide (CADB) as internal standard (IS).

Weighting/Dilution (Standard Solution)	CADB		Capsaicin RS		Dihydrocapsaicin RS	
	c (ng/ml)	Area	c (ng/ml)	Area	c (ng/ml)	Area
1/1	200	281.645	200	206.907	200	209.446
1/2	200	282.931	200	208.611	200	209.324
1/3	200	284.115	200	207.578	200	209.066
2/1	200	297.110	200	219.010	200	210.680
2/2	200	298.515	200	220.277	200	211.036
2/3	200	299.517	200	218.821	200	211.225
**Mean**		**290.639**		**213.534**		**210.130**
**RSD %**		**2.942**		**3.013**		**0.455**

**Table 5:** Intermediate precision data for 200 ng/mL acetonitrile solutions of Capsaicin RS, Dihydrocapsaicin RS, and internal standard (CADB).

Day	Dilution (Standard Solution)	CADB	Capsaicin RS	Dihydrocapsaicin RS
		Area	Area	Area
1	1.	282.931	208.611	209.324
	2.	284.115	207.578	209.066
	3.	298.515	220.277	211.036
				
2	1.	280.698	207.174	207.810
	2.	281.373	206.958	206.943
	3.	295.956	217.797	210.953
				
3	1.	284.195	211.165	209.74
	2.	286.657	210.898	210.037
	3.	299.469	221.129	215.672
**Mean**		**288.212**	**212.399**	**210.065**
**RSD %**		**2.629**	**2.713**	**1.186**

**Table 6:** Matrix effect of blank dog plasma extract dissolved in acetonitrile solutions containing Capsaicin RS (20, 100, 200, and 400 ng/mL), Dihydrocapsaicin RS (20, 100, 200, and 400 ng/mL) and internal standard (CADB) (c = 200 ng/mL).

CADB		Capsaicin RS		Dihydrocapsaicin RS	
c (ng/mL)	Area	c (ng/mL)	Area	c (ng/mL)	Area
200	309.861	20	17.580	20	18.341
200	309.816	20	18.680	20	18.181
200	307.434	20	17.852	20	18.770
200	310.107	20	18.329	20	18.086
200	304.773	100	93.517	100	95.614
200	310.350	100	93.559	100	96.691
200	319.699	100	96.824	100	98.475
200	313.438	100	93.435	100	98.698
200	309.791	200	181.260	200	193.176
200	310.974	200	193.205	200	211.009
200	309.756	200	190.162	200	203.587
200	315.218	200	187.609	200	205.103
200	320.154	400	390.450	400	384.022
200	311.768	400	380.979	400	387.807
200	329.947	400	393.093	400	404.482
200	334.889	400	406.833	400	402.899

**Table 7:** ANOVA results of matrix effect of blank dog plasma extract on acetonitrile solutions containing Capsaicin RS (20, 100, 200, and 400 ng/mL), Dihydrocapsaicin RS (20, 100, 200, and 400 ng/mL) and internal standard (CADB) (c = 200 ng/mL).

Compounds	c (ng/mL)	F_calculated_	F_critical_
CADB	200	4.3096	4.4139
Capsaicin RS	20	4.0824	5.9874
Capsaicin RS	100	4.7856	5.9874
Capsaicin RS	200	2.8410	5.9874
Capsaicin RS	400	0.0702	5.9874
Dihydrocapsaicin RS	20	0.3378	5.9874
Dihydrocapsaicin RS	100	0.7692	5.9874
Dihydrocapsaicin RS	200	0.2914	5.9874
Dihydrocapsaicin RS	400	3.0580	5.9874

## Supplementary Materials

The following are available online, Figure S1: HPLC-FLD chromatogram of the extract of the plasma of an experimental dog 0 min before *per os* administration of Capsaicin Natural (USP 29, 0.9 mg/kg) test item, spiked with cyclohexanecarboxylic acid-3,4-dimethoxybenzylamide (CADB) as internal standard (IS) (c = 20 ng/mL).; Figure S2: HPLC-FLD chromatogram of the extract of the plasma of an experimental dog 15 min after *per os* administration of Capsaicin Natural (USP 29, 0.9 mg/kg) test item, spiked with cyclohexanecarboxylic acid-3,4-dimethoxybenzylamide (CADB) as internal standard (IS) (c = 20 ng/mL).; Figure S3: HPLC-FLD chromatogram of the extract of the plasma of an experimental dog 30 min after *per os* administration of Capsaicin Natural (USP 29, 0.9 mg/kg) test item, spiked with cyclohexanecarboxylic acid-3,4-dimethoxybenzylamide (CADB) as internal standard (IS) (c = 20 ng/mL).; Figure S4: HPLC-FLD chromatogram of the extract of the plasma of an experimental dog 60 min after *per os* administration of Capsaicin Natural (USP 29, 0.9 mg/kg) test item, spiked with cyclohexanecarboxylic acid-3,4-dimethoxybenzylamide (CADB) as internal standard (IS) (c = 20 ng/mL).; Figure S5: HPLC-FLD chromatogram of the extract of the plasma of an experimental dog 120 min after *per os* administration of Capsaicin Natural (USP 29, 0.9 mg/kg) test item, spiked with cyclohexanecarboxylic acid-3,4-dimethoxybenzylamide (CADB) as internal standard (IS) (c = 20 ng/mL).; Figure S6: HPLC-FLD chromatogram of the extract of the plasma of an experimental dog 180 min after *per os* administration of Capsaicin Natural (USP 29, 0.9 mg/kg) test item, spiked with cyclohexanecarboxylic acid-3,4-dimethoxybenzylamide (CADB) as internal standard (IS) (c = 20 ng/mL).; Figure S7: HPLC-FLD chromatogram of the extract of the plasma of an experimental dog 240 min after *per os* administration of Capsaicin Natural (USP 29, 0.9 mg/kg) test item, spiked with cyclohexanecarboxylic acid-3,4-dimethoxybenzylamide (CADB) as internal standard (IS) (c = 20 ng/mL).; Figure S8: HPLC-FLD chromatogram of the extract of the plasma of an experimental dog 0 min before *per os* administration of Capsaicin Natural (USP 29, 4.8 mg/kg) test item, spiked with cyclohexanecarboxylic acid-3,4-dimethoxybenzylamide (CADB) as internal standard (IS) (c = 20 ng/mL).; Figure S9: HPLC-FLD chromatogram of the extract of the plasma of an experimental dog 120 min after *per os* administration of Capsaicin Natural (USP 29, 4.8 mg/kg) test item, spiked with cyclohexanecarboxylic acid-3,4-dimethoxybenzylamide (CADB) as internal standard (IS) (c = 20 ng/mL).
