# Supplementary material for: Toxicokinetic Study of a Gastroprotective Dose of Capsaicin by HPLC-FLD Method
Source: Molecules. 2019 Aug 5;24(15):2848. doi: 10.3390/molecules24152848 (PMC6695877; doi:10.3390/molecules24152848)
Supplement: Supplementary file 1 [file molecules-24-02848-s001.pdf]

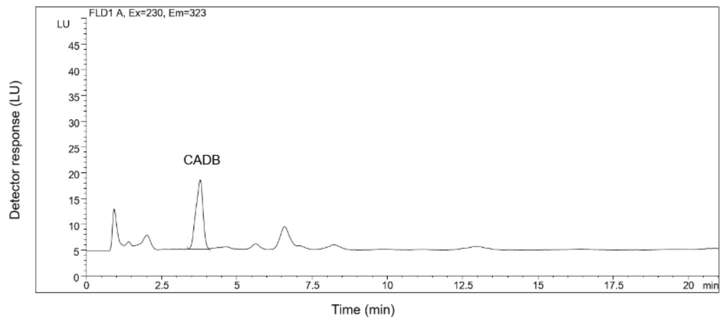

Figure S1. Chromatogram of the extract of the plasma of an experimental dog 0 min before *per os* administration of Capsaicin natural (USP 29, 0.9 mg/kg) test item, spiked with cyclohexanecarboxylic acid-3,4-dimethoxybenzylamide (CADB) as internal standard (IS) (c=20 ng/mL).

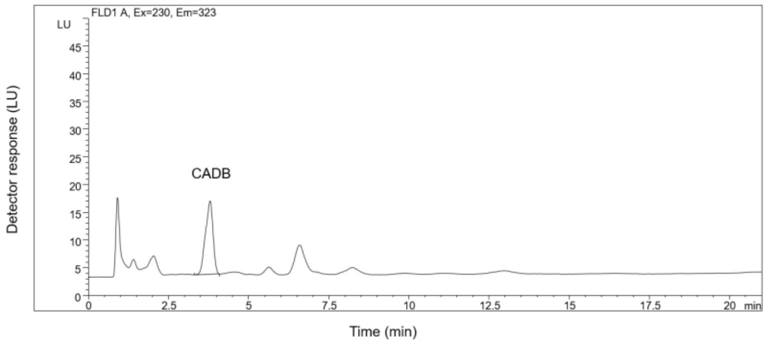

Figure S2. Chromatogram of the extract of the plasma of an experimental dog 15 min after *per os* administration of Capsaicin natural (USP 29, 0.9 mg/kg) test item, spiked with cyclohexanecarboxylic acid-3,4-dimethoxybenzylamide (CADB) as internal standard (IS) (c=20 ng/mL).

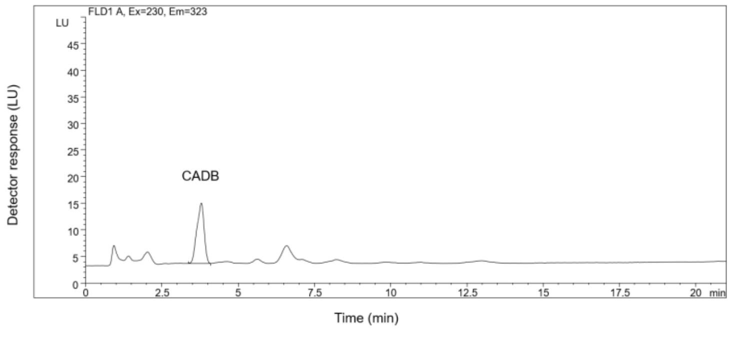

Figure S3. Chromatogram of the extract of the plasma of an experimental dog 30 min after *per os* administration of Capsaicin natural (USP 29, 0.9 mg/kg) test item, spiked with cyclohexanecarboxylic acid-3,4-dimethoxybenzylamide (CADB) as internal standard (IS) (c=20 ng/mL).

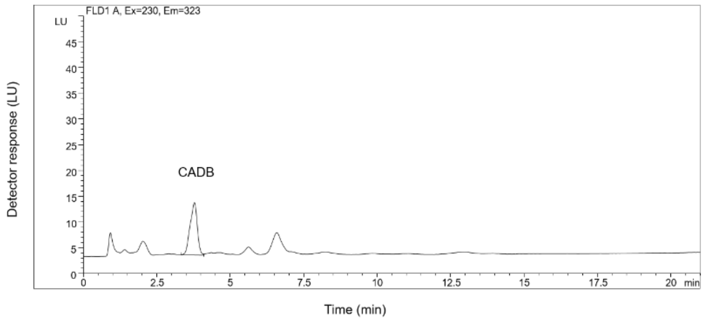

Figure S4. Chromatogram of the extract of the plasma of an experimental dog 60 min after *per os* administration of Capsaicin natural (USP 29, 0.9 mg/kg) test item, spiked with cyclohexanecarboxylic acid-3,4-dimethoxybenzylamide (CADB) as internal standard (IS) (c=20 ng/mL).

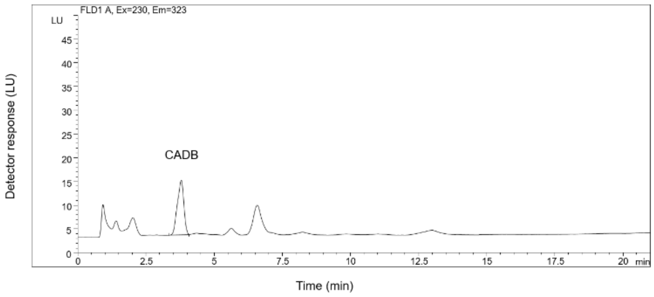

Figure S5. Chromatogram of the extract of the plasma of an experimental dog 120 min after *per os* administration of Capsaicin natural (USP 29, 0.9 mg/kg) test item, spiked with cyclohexanecarboxylic acid-3,4-dimethoxybenzylamide (CADB) as internal standard (IS) (c=20 ng/mL).

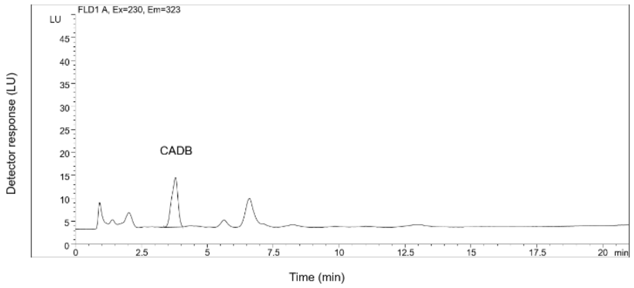

Figure S6. Chromatogram of the extract of the plasma of an experimental dog 180 min after *per os* administration of Capsaicin natural (USP 29, 0.9 mg/kg) test item, spiked with cyclohexanecarboxylic acid-3,4-dimethoxybenzylamide (CADB) as internal standard (IS) (c=20 ng/mL).

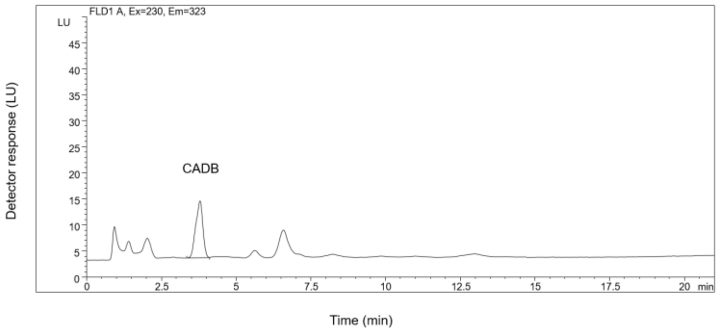

Figure S7. Chromatogram of the extract of the plasma of an experimental dog 240 min after *per os* administration of Capsaicin natural (USP 29, 0.9 mg/kg) test item, spiked with cyclohexanecarboxylic acid-3,4-dimethoxybenzylamide (CADB) as internal standard (IS) (c=20 ng/mL).

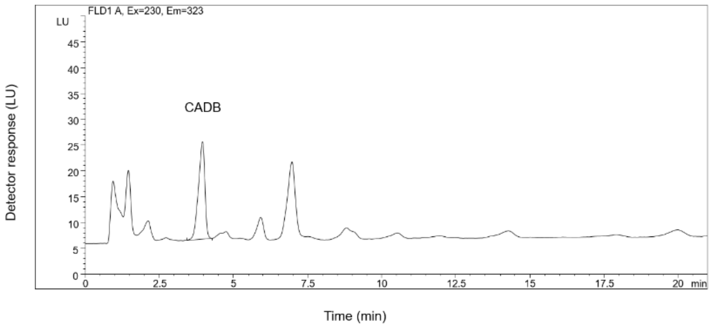

Figure S8. Chromatogram of the extract of the plasma of an experimental dog 0 min before *per os* administration of Capsaicin natural (USP 29, 4.8 mg/kg) test item, spiked with cyclohexanecarboxylic acid-3,4-dimethoxybenzylamide (CADB) as internal standard (IS) (c=20 ng/mL).

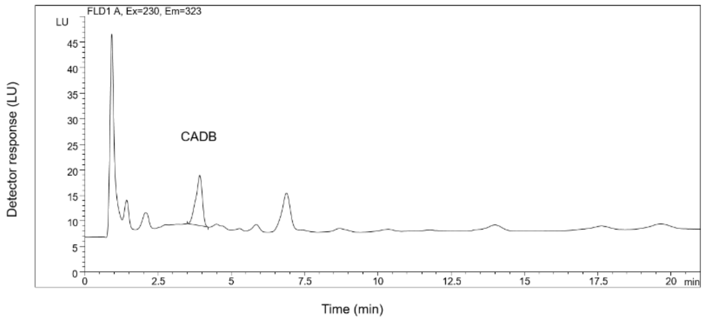

Figure S9. Chromatogram of the extract of the plasma of an experimental dog 120 min after *per os* administration of Capsaicin natural (USP 29, 4.8 mg/kg) test item, spiked with cyclohexanecarboxylic acid-3,4-dimethoxybenzylamide (CADB) as internal standard (IS) (c=20 ng/mL).
